# Characteristics and Research Waste Among Randomized Clinical Trials in Gastric Cancer

**DOI:** 10.1001/jamanetworkopen.2021.24760

**Published:** 2021-09-17

**Authors:** Jun Lu, Bin-bin Xu, Li-li Shen, Dong Wu, Zhen Xue, Hua-Long Zheng, Jian-Wei Xie, Jia-Bin Wang, Jian-Xian Lin, Qi-Yue Chen, Long-Long Cao, Mi Lin, Ru-Hong Tu, Ze-Ning Huang, Ju-Li Lin, Chang-Ming Huang, Chao-Hui Zheng, Ping Li

**Affiliations:** 1 Department of Gastric Surgery, Fujian Medical University Union Hospital, Fuzhou, China; 2 Department of General Surgery, Fujian Medical University Union Hospital, Fuzhou, China; 3 Key Laboratory of Ministry of Education of Gastrointestinal Cancer, Fujian Medical University, Fuzhou, China; 4 Fujian Key Laboratory of Tumor Microbiology, Fujian Medical University, Fuzhou, China

## Abstract

**Importance:**

The results of numerous large randomized clinical trials (RCTs) have changed clinical practice in gastric cancer (GC). However, research waste (ie, unpublished data, inadequate reporting, or avoidable design limitations) is still a major challenge for evidence-based medicine.

**Objectives:**

To determine the characteristics of GC RCTs in the past 20 years and the presence of research waste and to explore potential targets for improvement.

**Design, Setting, and Participants:**

In this cross-sectional study of GC RCTs, ClinicalTrials.gov was searched for phase 3 or 4 RCTs registered from January 2000 to December 2019 using the keyword *gastric cancer*. Independent investigators undertook assessments and resolved discrepancies via consensus. Data were analyzed from August through December 2020.

**Main Outcomes and Measures:**

The primary outcomes were descriptions of the characteristics of GC RCTs and the proportion of studies with signs of research waste. Research waste was defined as unpublished data, inadequate reporting, or avoidable design limitations. Publication status was determined by searching PubMed and Scopus databases. The adequacy of reporting was evaluated using the Consolidated Standards of Reporting Trials (CONSORT) reporting guideline checklist. Avoidable design limitations were determined based on existing bias or lack of cited systematic literature reviews. In the analyses of research waste, 125 RCTs that ended after June 2016 without publication were excluded.

**Results:**

A total of 262 GC RCTs were included. The number of RCTs increased from 25 trials in 2000 to 2004 to 97 trials in 2015 to 2019, with a greater increase among RCTs of targeted therapy or immunotherapy, which increased from 0 trials in 2000 to 2004 to 36 trials in 2015 to 2019. The proportion of RCTs that were multicenter was higher in non-Asian regions than in Asian regions (50 of 71 RCTs [70.4%] vs 96 of 191 RCTs [50.3%]; *P* = .004). The analysis of research waste included 137 RCTs, of which 81 (59.1%) were published. Among published RCTs, 65 (80.2%) were judged to be adequately reported and 63 (77.8%) had avoidable design defects. Additionally, 119 RCTs (86.9%) had 1 or more features of research waste. Study settings that included blinding (odds ratio [OR], 0.56; 95% CI, 0.33-0.93; *P* = .03), a greater number of participants (ie, ≥200 participants; OR, 0.07; 95% CI, 0.01-0.51; *P* = .01), and external funding support (OR, 0.22; 95% CI, 0.08-0.60; *P* = .004) were associated with lower odds of research waste. Additionally, 35 RCTs (49.3%) were referenced in guidelines, and 18 RCTs (22.2%) had their prospective data reused.

**Conclusions and Relevance:**

To our knowledge, this study is the first to describe the characteristics of GC RCTs in the past 20 years, and it found a research waste burden, which may provide evidence for the development of rational RCTs and reduction of waste in the future.

## Key Points

**Question:**

Is there research waste (ie, unpublished data, inadequate reporting, or avoidable design limitations) in randomized clinical trials (RCTs) of gastric cancer?

**Findings:**

This cross-sectional study included 137 RCTs, of which 119 had 1 or more features of research waste. Additionally, 35 RCTs were referenced in guidelines, and 18 had their prospective data reused.

**Meaning:**

This study found a research waste burden in gastric cancer RCTs during the past 20 years, which may provide evidence for the development of rational RCTs and reduction of waste in the future.

## Teaser

This cross-sectional study examines the characteristics and presence of research waste, defined as unpublished data, inadequate reporting, or avoidable design limitations, among randomized clinical trials in gastric cancer.

## Introduction

Gastric cancer (GC) is the fifth most common human malignant tumor, ranking third in cancer-related mortality.^1^ More than 1 million GC diagnoses are made globally each year.^2^ To improve the prognosis of patients with GC, increased numbers of randomized clinical trials (RCTs) have been carried out to explore new and potentially more effective treatments. In the past 20 years, these RCTs have provided high-level medical evidence for the clinical diagnosis and treatment of GC. Based on these findings, postoperative recovery and long-term survival of patients with GC have improved significantly.^3,4,5,6^

Although RCTs provide high-level evidence and have made important contributions to the advancement of treatment, research waste is inevitably a major challenge to evidence-based medicine. This means that wasteful RCTs used resources while the risks to participants increased. Chapman et al^7^ found that 85.2% of surgical-related RCTs had research waste. This waste may occur at any stage of the research cycle. First, RCTs may be wasteful owing to avoidable design flaws. For example, improper implementation of randomization or blindness procedures is associated with decreased credibility of research results. Waste may also be associated with a failure to consider previous evidence. Second, if results were not published for any reason, the research was also wasted. Third, published RCTs may be considered wasteful owing to insufficient reporting, making it difficult or impossible to use and repeat research reports. Research wastefulness may ultimately mean that RCT results cannot be adopted in the clinical practice guidelines of the relevant field.

Currently, to our knowledge, research waste has not been explored for GC RCTs. With increasing incidence of treatment-resistant GC subtypes,^8^ it is important to develop more RCTs aimed at the development of new and effective treatments for GC. Minimizing research waste is essential to ensure that new treatments are appropriately, safely, and effectively translated into clinical practice.

We completed one of the most comprehensive analyses to date of GC RCTs, focusing on characteristics and components of research waste among GC RCTs in the past 20 years to identify potential targets for improvement. In addition, we explored whether published RCTs were referenced in guidelines and whether relevant prospective data were reused.

## Methods

Fujian Medical University Union Hospital Ethics Committee determined that because this cross-sectional study was a review of registered RCTs, it was exempt from research ethics approval and the requirement for informed consent. This study followed the Strengthening the Reporting of Observational Studies in Epidemiology (STROBE) reporting guideline.

### Design and Data Sources

The data used in this study were derived from ClinicalTrials.gov, a publicly available online trial registry.^9^ We searched the ClinicalTrials.gov database using the keyword *gastric cancer* on a single day (March 1, 2020). Eligible trials were phase 3 or 4 trials conducted from January 1, 2000, to December 3, 2019. Titles and trial abstracts were screened to determine study eligibility. Studies were excluded if they were not randomized trials, not phase 3 or phase 4 trials, not related to GC, or duplicated. Independent investigators (M.L. and J.-B. W.) undertook assessments and resolved discrepancies through consensus. Basic characteristics of RCTs were derived from ClinicalTrials.gov, including study interventions, funding source (ie, industry funding, other external funding, or no funding or departmental funding), and other key elements audited by ClinicalTrials.gov review staff.^10^ We divided funding sources into (1) none or departmental funding or (2) industry or other external funding. To better present the development of RCTs, we divided RCTs into 4 focus categories: (1) perioperative chemoradiotherapy, which did not include targeted therapy or immunotherapy; (2) targeted therapy or immunotherapy; (3) surgical treatment; and (4) other. Results from previous studies on the cutoff value for sample size were not uniform, and there is no specific definition for this value.^7,11,12,13,14,15,16^ In this study, we predefined RCTs with sample sizes smaller than the smallest 25% samples as small-sample RCTs. Because the incidence of GC is highly variable by region and culture, with higher incidence rates overall in Asian countries, we classified RCTs into Asian and non-Asian trials based on the location of the principal investigator (PI). A positive RCT was defined as an RCT in which the primary end point had a statistically significant difference in favor of the experimental group.^17^

### Status of Publication

To determine publication status, we performed searches using the ClinicalTrials.gov identifier (National Clinical Trial [NCT] number), name of the PI, and several keywords related to the RCT on PubMed and Scopus. We then checked the manuscript’s information, including interventions, study population, and dates of recruitment, to further confirm publication status. If no corresponding manuscripts from PubMed and Scopus were available, we contacted the corresponding PI to further determine publication status. When no response was received, the RCT was determined to be unpublished by default. A trial was considered to be published if a full-text manuscript (available in print or online) was identified in a peer-reviewed journal.^7^ The search was performed by 2 independent investigators (M.L. and J.-B. W.). The last search was conducted on June 30, 2020.

### Reporting Adequacy Assessment

The assessment of the adequacy of reporting for each manuscript was performed using the Consolidated Standards of Reporting Trials (CONSORT) reporting guideline statement.^18,19^ There were 37 items for each manuscript involving pharmacological interventions and 40 items for each manuscript involving nonpharmacological interventions.^18,19^ Therefore, to conform to the CONSORT statement, we divided RCTs into pharmacological interventions and nonpharmacological interventions, with 1 point added to the reporting adequacy score for each item. A single investigator (J.-X.L.) downloaded all manuscripts and supplementary correspondence materials. Then, using Adobe Acrobat Pro PDF software version XI Pro (Adobe), which could transform PDF documents into Word documents, the single investigator deleted the author and journal information and reformatted the text to reduce biases. Finally, the files were printed and 2 independent investigators (B.-B.X. and H.-L.Z.) reviewed and scored all manuscripts independently according to the CONSORT 2010 checklist. Discrepancies were discussed and resolved through consensus after every 3 manuscripts reviewed. Because the CONSORT reporting guideline statement has many items and each item was assigned a point, the interrater agreement was not recorded. The full-text manuscript and correspondence supplementary materials were reviewed to determine whether a protocol was available.^7^ Manuscripts that met 75% of items (ie, 28 of 37 pharmacological items or 30 of 40 nonpharmacological items) were considered to be reported adequately, as previously described.^7^

### Design Flaw Assessment

Using the Cochrane tool,^20^ 2 independent investigators (B.-B.X. and H.-L.Z.) reviewed masked manuscripts and assessed the risk of bias, including selection bias, performance bias, detection bias, attrition bias, reporting bias, and other biases. Each item was determined to be low risk, unclear, or high risk. Discrepancies were discussed and resolved through consensus after every 3 manuscripts reviewed. Items with an unclear risk of bias were considered to be high-risk items in the statistical analyses. This was done because unclear descriptions of key methods are associated with judgments of an RCT’s ability to inform practice. Additionally, the presence of a relevant systematic review, or justification as to why one was not necessary in novel settings, was also assessed. This had to be cited in the full-text manuscript and be considered capable of informing the necessity of carrying out the RCT to be counted as present. Articles that presented 1 of the previously listed biases or lacked a relevant systematic review citation were considered to have an avoidable design flaw.

### Whether Referenced in Guidelines and Reuse of Prospective Data

For each published RCT, we first searched articles that cited the published RCT in the Google Scholar databases.^21^ Subsequently, 2 independent investigators (B.-B.X. and H.-L.Z.) manually checked each article that referred to the RCT to determine whether treatment or practice guidelines were present. We also assessed whether prospective data were reused for post hoc analysis (ie, data from the RCT were reanalyzed for outcomes other than preset primary or secondary end points).^22,23,24^ We assumed that the post hoc analysis always cited the original RCT.

### Outcomes

The primary outcomes were to elaborate characteristics of RCTs in the past 20 years and to explore research waste (ie, nonpublication, inadequate reporting, and avoidable design flaws). The secondary outcomes were to explore potentially modifiable research waste. Additionally, we determined whether the published RCTs were referenced in guidelines and explored the reuse of prospective data. Publication of an RCT is the precondition for assessments of report adequacy and design flaws. Therefore, in the analyses of research waste, we included all published RCTs and excluded RCTs completed after June 2016 without publication.

### Statistical Analysis

Differences between groups in categorical variables were compared using the χ^2^ or Fisher test (if the number of samples was <5).^25^ A second-order polynomial equation was fitted to the data by a multiple regression method. Simple and multivariable logistic regression models were used to determine independent risk factors associated with research waste. Variables with a value of *P* < .05 in the simple analysis were subsequently included in a multivariable analysis. All statistical analyses were performed using SPSS statistical software version 18.0 for Windows (IBM) and R statistical software version 4.0.2 (R Project for Statistical Computing). *P* values < .05 were considered statistically significant, and all tests were 2-sided. Data were analyzed from August through December 2020.

## Results

### Characteristics of RCTs in the Past 20 Years

From January 2000 to December 2019, there were 306 clinical trials that met inclusion criteria that were retrieved. After we excluded 33 nonrandomized clinical trials, 3 trials that were not phase 3 or 4 trials, 9 trials not related to GC, and 1 duplicated trial, 262 GC RCTs were included in the analysis. Most RCTs were pharmacologically related (191 trials [72.9%]), and most RCTs had PIs who were located in Asia (191 trials [72.9%]). More RCTs were multicenter than single center (146 trials [55.7%] vs 116 trials [44.3%]). The median (interquartile range) sample size of RCTs was 394 (186-579) participants. Therefore, we defined the RCTs with sample sizes of less than 200 individuals as small RCTs. There were 77 RCTs (29.4%) with external funding (Table 1).

**Table 1.** Characteristics of Included RCTs

Characteristic	RCTs, No. (%) (N = 262)
Year of registration	
2000-2004	25 (9.5)
2005-2009	43 (16.4)
2010-2014	97 (37.0)
2015-2019	97 (37.0)
Intervention	
Pharmacological	191 (72.9)
Nonpharmacological	71 (27.1)
Study design	
Parallel group	261 (99.6)
Factorial	1 (0.4)
Phase	
3	235 (89.7)
4	27 (10.3)
Region of PI	
Asian	191 (72.9)
Non-Asian	71 (27.1)
Study groups, No.	
2	245 (93.5)
3	12 (4.6)
≥4	5 (1.9)
Blinding	
None or open label	180 (68.7)
Single	18 (6.9)
Double	27 (10.3)
Triple	13 (5.0)
Quadruple	24 (9.2)
Recruitment	
Monocentric	116 (44.3)
Multicenter	146 (55.7)
Participants, No.	
<100	51 (19.5)
≥100	211 (80.5)
<200	87 (33.2)
≥200	175 (66.8)
Funding	
None or departmental	185 (70.6)
Industry or other external	77 (29.4)

Abbreviations: PI, principal investigator; RCT, randomized clinical trial.

The number of GC RCTs increased from 1 phase 3 RCT in 2000 to 30 phase 3 and 4 RCTs in 2015 and then flattened out to 15 RCTs per year in 2018 and 2019. There were 25 trials in 2000 to 2004 and 97 trials in 2015 to 2019. The proportion of RCTs related to perioperative chemoradiotherapy decreased continuously, from 13 of 25 trials (52.0%) to 29 of 97 trials (29.9%). In contrast, the number and proportion of RCTs related to targeted therapy or immunotherapy increased from 0 of 25 trials in 2000 to 2004 to 36 of 97 trials (37.1%) in 2015 to 2019. The number of RCTs of surgical treatments and other RCTs had smaller increases over the past 20 years, changing from 3 of 25 trials (12.0%) to 15 of 97 trials (15.5%) and from 9 of 25 trials (36.0%) to 17 of 97 trials (17.5%), respectively (Figure).

**Figure.**
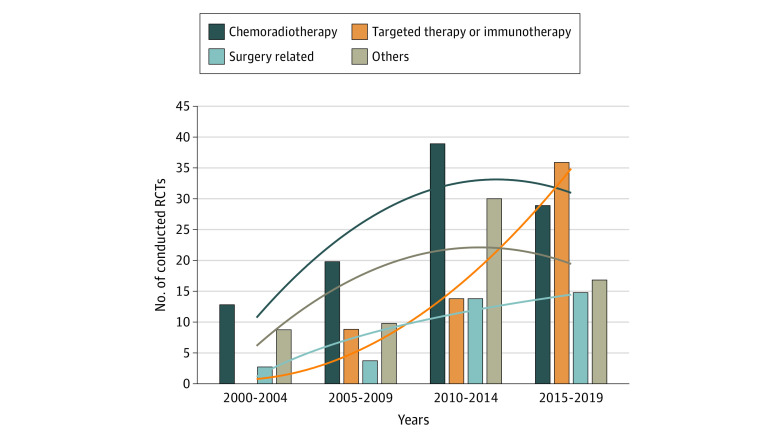
Number of Conducted Randomized Clinical Trials (RCTs) by Category Lines indicate trend lines for associated RCT categories.

There were more RCTs in Asia than in non-Asian regions (191 RCTs [72.9%] vs 71 RCTs [27.1%]). However, the proportion of multicenter RCTs in non-Asian regions was higher than that in Asian regions (50 trials [70.4%] vs 96 trials [50.3%]; *P* = .004) (eFigure 1 in the Supplement).

### Nonpublication

After we excluded 125 RCTs completed after June 2016 without publication, there were 137 completed RCTs, among which 81 trials (59.1%) were published with the full text available for review and 56 trials (40.9%) were unpublished. The proportion of unpublished RCTs from Asia was greater than that from non-Asian regions (47 trials [83.9%] vs 9 trials [16.1%]; *P* = .02). In addition, unpublished RCTs, compared with published RCTs, were more likely to have a smaller sample size (ie, <200 participants) (34 trials [60.7%] vs 23 trials [28.4%]; *P* < .001) and a single-center design (41 trials [73.2%] vs 23 trials [28.4%]; *P* < .001) and less likely to have external funding (7 trials [12.5%] vs 34 trials [42.0%]; *P* < .001). Other characteristics related to RCT design were comparable between the 2 groups (Table 2). In logistic regression analysis of the association of key study characteristics with publication status, RCTs developed in non-Asian regions or with multicenter designs or external funds were more likely to be published (eTable 1 in the Supplement).

**Table 2.** Characteristics of RCTs Completed Before June 2016 by Publication Status

Characteristic	Completed RCTs, No. (%) (N = 137)		*P* value
	Published (n= 81)	Not published (n = 56)	
Year of registration			
2000-2009	47 (58.0)	20 (35.7)	.01
After 2009	34 (42.0)	36 (64.3)	
Study groups, No.			
2	78 (96.3)	49 (87.5)	.09
≥3	3 (3.7)	7 (12.5)	
Region of PI			
Asian	53 (65.4)	47 (83.9)	.02
Non-Asian	28 (34.6)	9 (16.1)	
Blinding			
None or open label	50 (61.7)	37 (66.1)	.35
Single	6 (7.4)	7 (12.5)	
Double or more	25 (30.9)	12 (21.4)	
Recruitment			
Monocentric	23 (28.4)	41 (73.2)	<.001
Multicenter	58 (71.6)	15 (26.8)	
Participants, No.			
<200	23 (28.4)	34 (60.7)	<.001
≥200	58 (71.6)	22 (39.3)	
Funding			
None or departmental	47 (58.0)	49 (87.5)	<.001
Industry or other external	34 (42.0)	7 (12.5)	

Abbreviations: PI, principal investigator; RCT, randomized clinical trial.

### Adequacy of Reporting

The scores of 81 published RCTs according to the CONSORT checklist are shown in eTable 2 in the Supplement. Of 62 RCTs with a drug intervention, the reported flaws that occurred most frequently involved the availability of the study protocol (44 trials [68.7%]), random sequence generation mechanism (35 trials [54.7%]), and random sequence allocation implementation (34 trials [53.1%]). Among 17 RCTs with a nonpharmacological intervention, the reported deficiencies most frequently involved the evaluation of adherence to the treatment protocol (16 trials [94.1%]), standardization of the intervention (13 trials [76.5%]), availability of study protocol (12 trials [70.6%]), random sequence generation mechanism (11 trials [64.7%]), and random sequence allocation implementation (11 trials [64.7%]). In total, 65 RCTs (80.2%) were judged to be adequately reported, and 16 RCTs (19.8%) were judged to be inadequately reported. Adequately reported RCTs, compared with inadequately reported RCTs, were more likely to be for pharmacological interventions (56 trials [86.2%] vs 8 trials [50.0%]; *P* = .004), have multicenter designs (50 trials [76.9%] vs 8 trials [50.0%]; *P* = .03), and include more patients (ie, ≥200 patients; 51 trials [78.5%] vs 7 trials [43.8%]; *P* = .01) (eTable 3 in the Supplement).

### Design Flaws

Among 81 RCTs that were published, 26 RCTs (32.1%) lacked a cited systematic review in the text and 63 RCTs (77.8%) had 1 or more features that indicated a high or unclear risk of bias. The most common factors associated with the risk of bias were selective reporting (51 trials [63.0%]), random sequence allocation concealment (46 trials [56.8%]), and assessor blinding (44 trials [54.3%]) (eFigure 2 in the Supplement). Considering all of these factors, 63 RCTs (77.8%) were judged to have avoidable design defects. These RCTs were more likely to be registered earlier (ie, between 2000 and 2009; 41 trials [65.1%] vs 6 trials [33.3%]; *P* = .02) and have fewer patients included (ie, <200 patients; 11 trials [17.5%] vs 1 trial [5.6%]; *P* = .02) compared with 18 trials without avoidable design defects (eTable 4 in the Supplement).

### Research Waste

Considering the composite of publication status, sufficient reporting, and avoidable design flaws, 119 of 137 RCTs (86.9%) had 1 or more features of research waste. These RCTs, compared with 18 RCTs without research waste, were more likely to have nonpharmacological interventions (34 trials [28.6%] vs 1 trial [5.6%]; *P* = .04), open label designs (80 trials [67.2%] vs 7 trials [38.9%]; *P* = .04), single-center designs (61 trials [51.3%] vs 3 trials [16.7%]; *P* = .006), smaller sample sizes (ie, <200 participants; 56 trials [47.1%] vs 1 trial [5.6%]; *P* = .001), and no external funding (89 trials [74.8%] vs 7 trials [38.9%]; *P* = .002) (Table 3). In further analysis, study settings that included blindness (odds ratio [OR], 0.56; 95% CI, 0.33-0.93; *P* = .03), a greater number of participants (ie, ≥200 participants; OR, 0.07; 95% CI, 0.01-0.51; *P* = .01), and external funding support (OR, 0.22; 95% CI, 0.08-0.60; *P* = .004) were associated with lower odds of research waste (Table 4).

**Table 3.** Characteristics of RCTs by Presence of Research Waste

Characteristic	Completed RCTs, No. (%) (N = 137)		*P* value
	Without research waste (n = 18)	With research waste (n = 119)	
Year of registration			
2000-2009	6 (33.3)	61 (51.3)	.16
After 2009	12 (66.7)	58 (48.7)	
Phase			
3	17 (94.4)	98 (82.4)	.19
4	1 (5.6)	21 (17.6)	
Intervention			
Pharmacological	17 (94.4)	85 (71.4)	.04
Nonpharmacological	1 (5.6)	34 (28.6)	
Region of PI			
Asian	11 (61.1)	89 (74.8)	.22
Non-Asian	7 (38.9)	30 (25.2)	
Study groups, No.			
2	18 (100)	109 (91.6)	.36
≥3	0	10 (8.4)	
Blinding			
None or open label	7 (38.9)	80 (67.2)	.04
Single	2 (11.1)	11 (9.2)	
Double or more	9 (50.0)	28 (23.5)	
Recruitment			
Monocentric	3 (16.7)	61 (51.3)	.006
Multicenter	15 (83.3)	58 (48.7)	
Participants, No.			
<200	1 (5.6)	56 (47.1)	.001
≥200	17 (94.4)	63 (52.9)	
Funding			
None or departmental	7 (38.9)	89 (74.8)	.002
Industry or other external	11 (61.1)	30 (25.2)	

Abbreviations: PI, principal investigator; RCT, randomized clinical trial.

**Table 4.** Association of Key Study Characteristics With Research Waste

Characteristic	Univariate analysis	
	OR (95% CI)	*P* value
Region of PI		
Asian	1 [Reference]	NA
Non-Asian	0.53 (0.19-1.49)	.23
Blinding		
None or open label	1 [Reference]	NA
Single or more	0.56 (0.33-0.93)	.03
No. of participants		
<200	1 [Reference]	NA
≥200	0.07 (0.01-0.51)	.01
Funding		
None or departmental	1 [Reference]	NA
Industry or other external	0.22 (0.08-0.60)	.004

Abbreviations: NA, not applicable; OR, odds ratio; PI, principal investigator.

### Referenced in Guidelines and Reuse of Prospective Data

Among 81 published RCTs, 50 trials (61.7%) reported positive results, and 31 RCTs (38.3%) reported negative results. We excluded 10 RCTs that were published in 2 recent years (ie, 2019 and 2020) and found that 35 trials (49.3%) were referenced in corresponding guidelines. Trials that included 200 participants or more and reported positive results were more likely to be referenced in guidelines (eTables 5 and 6 in the Supplement). In addition, 18 RCTs (22.2%) had prospective data that were reused. Trials developed in non-Asian regions or that had multicenter designs or positive results were more likely to have prospective data that were reused (eTables 7 and 8 in the Supplement).

## Discussion

In this cross-sectional study, the characteristics of 262 GC RCTs in the past 20 years were analyzed for the first time, to our knowledge, and considerable research waste was found (86.9% of RCTs had ≥1 feature of research waste). Of 137 RCTs completed before June 2016, 81 RCTs (59.1%) were published. Moreover, 63 RCTs (77.8%) were judged to have avoidable design defects. In addition, 35 RCTs (49.3%) were referenced in corresponding guidelines. In further analyses, double-blind study design, a greater number of patients included, and external funding were associated with decreased odds of research waste.

The best way to minimize bias when evaluating novel treatments in health care is through RCTs.^26^ The degree of disease burden determines which RCTs should be performed to improve the situation. However, previous studies^27,28^ found a mismatch between disease burden and research funding. In 2001, a major research funder of the UK Department of Health and Social Care emphasized that a systematic review of previous related research and existing evidence is essential when deciding on further research.^29^ New research should not be conducted unless the questions to be resolved at the beginning cannot be answered satisfactorily with existing evidence. We conducted analyses of the characteristics of GC RCTs carried out in the past 20 years and found that the number of RCTs increased by year and then the trend stabilized. This also suggests that the phenomenon of research waste deserves attention. When high-level medical evidence for surgical treatment, especially minimally invasive surgical treatment for patients with GC, has been established,^30,31^ it is not difficult to speculate that systemic treatment will comprise the main research direction in the future. We also found that the proportion of systematic treatment was as high as 67% in the past 5 years, with greater proportions for targeted therapy or immunotherapy. Although this may be an important factor associated with whether breakthroughs can be made in the treatment of GC in the future, attention should be paid to designing RCTs that are compatible with the latest results and development directions to avoid research waste.

Publication of RCTs can provide high-level evidence for practice guidelines for GC. In this study, we found that although there were more RCTs with PIs based in Asia than those with PIs based outside of Asian (which was expected), the latter had a higher proportion of multicenter RCTs and published RCTs than the former. Although these findings may be only statistically associated, the smaller number of GC diagnoses in non-Asian regions may be associated with the development of more multicenter RCTs, with an indirect association with the quality and publication rate of GC RCTs. These findings suggest that, in the future, scholars should strengthen the collaboration among multiple centers when designing RCTs, especially in Asia.

Additionally, to consider similar research conducted at the same time when designing novel research, design features may also be associated with the size of the effect estimate. For RCTs, allocation concealment, study blindness, and improper use of random allocation sequence methods may be associated with the effect estimate, especially when the study end point is a subjective result.^32^ Overall, this study found that 77.8% of RCTs had avoidable design flaws. Common design flaws included flaws in random sequence allocation concealment and evaluator blindness. Trials with design flaws were more likely to be registered earlier and have a smaller sample size. In addition, a lack of external funding was associated with design flaws.

The sufficiency of results reporting is critical for readers to judge whether research applies to their practice. In general, most RCTs (80.2%) were adequately reported. However, it is particularly noteworthy that reporting quality was unsatisfactory for study protocol and randomization. Approximately 30% of trials provided their study protocols. The lack of a study protocol may reduce readers' understanding of the entire RCT design. The unreasonable use of randomization methods may also be associated with potential bias. We additionally found that in surgical-related RCTs, there was a lack of detailed descriptions of surgical interventions and methods of intervention standardization. Unlike drug intervention, surgical intervention has a high degree of variability in methods, techniques, equipment, and prostheses used. The level of detail reported on intervention measures may be directly associated with the credibility and repeatability of the research conclusion.

Additionally, 119 RCTs (86.9%) had 1 or more features of research waste. In a multivariable analysis adjusting for other variables, the implementation of study blindness, a study population of 200 or more participants, and external funding support were associated with lower odds of research waste. Nevertheless, these attributes should not be equated with research waste itself. Because of the small sample size, small RCTs without external funding were the preliminary basis for future large RCTs, and these elements could enable scholars to initially understand relevant information in the shortest time. Instead, it is important to consider quality improvement programs that expand the basic availability of resources, such as statistics and test management. In addition, if these design features are associated with insufficient resources, it is particularly important to encourage cooperation among multiple centers and reduce repeated research to reduce unnecessary capital expenditures. Currently, this can be achieved through the participation of accredited clinical trial research departments or local support infrastructure, which can provide the necessary logistical and methodological support.

Trials funded by external manufacturers or industry were less likely to have research waste. The association between industry funding and research results is a popular topic, which is not limited to GC. However, protocol is important for investigators to judge whether an RCT result is improper (eg, selective reporting). The number of RCTs with an available study protocol was relatively small. More importantly, the specific research field of our study (GC and phase 3 or 4 RCTs) made it difficult to further explore the potential association of industry funding with research results. A further larger cross-sectional study is warranted.

Previous studies^7,33,34^ found that approximately 14% to 60% of all RCTS received industry funding. Additionally, Jairam et al^34^ found that among phase 3 oncology clinical trials, RCTs of targeted systemic agents were more likely to be externally funded. Del et al^11^ reported that, over time, there has been an increase in the proportion of RCTs with industry funding. Our analyses found that 77 RCTs (29.4%) were externally funded. Among 81 published RCTs, 34 trials (42.0%) were externally funded. These results reflected the real situation of GC RCTs over the past 20 years, but the underlying reasons were unclear. The relatively low proportion may be associated with the focus on GC and the study period.

Publication bias in medical journals refers to the publication of more articles containing positive conclusions or statistically significant results.^22^ Previous studies^17,22,35,36^ found that there is a time lag in the publication of negative findings and that studies with large sample sizes and positive results are often easier to publish, which is likely associated with publication bias. In our study, more than half of published RCTs reported positive results.

Additionally, this study explored the reuse of prospective data and whether published RCTs were referenced in guidelines. Among 81 RCTs that were published, 35 (49.3%) were referenced in guidelines. Trials with 200 participants or more and positive results were more likely to be referenced in guidelines. This may be expected because positive results seem to be more in line with the needs of clinical practice. In addition, Correa et al^37^ emphasized the advantages of prospective data and the importance of reusing these data. However, we found that among published RCTs, 18 (22.2%) had prospective data that were reused. Trials that had multicenter designs, were based in non-Asian regions, or had positive results were more likely to have their prospective data reused. We considered that having weak efficacy results (ie, negative results) may be a factor associated with the lack of prospective data reuse. The potential value of these negative data has yet to be exploited fully. This suggests that further efforts to make the best use of available data in combination with specific prospective data collection are warranted.

Conducting an RCT usually requires a great deal of labor and material and financial resources. Although there has been a similar report,^7^ this study is the first, to our knowledge, to focus on clinical trials with regard to a specific field (ie, GC). Most of the findings obtained are original, which may contribute to the proper conduct of clinical trials.

### Limitations

This study has several limitations. First, quantifying research waste is complex. Such waste is by no means limited to the 3 elements defined in this study. For example, repeated RCTs that explore low-priority research topics with clear medical evidence could also have research waste. Second, various end points were collected manually, which may be associated with measurement errors. However, we had 2 independent investigators evaluate each RCT and resolve differences through discussion so that measurement errors could be minimized as much as possible. Third, although ClinicalTrials.gov is a comprehensive clinical trial registry, which accounted for more than 60% of trials in the past 20 years,^38^ the World Health Organization has also recognized other country-specific and region-specific registries,^10^ and RCTs in those registries were not included in this study. Our study presented only a preliminary viewpoint for scientists. Fourth, this study could not further explore the potential reasons or mechanisms of the association of small sample sizes or lack of external funding support with research waste. This may be associated with the difficulty in obtaining effective infrastructure support when designing RCTs, which is associated with research waste. Additionally, the 2 CONSORT checklists used in this study were published in 2010 and were also applied to RCTs published before 2010. Nevertheless, we believe that this had a limited association with study outcomes because these 2 updates contained the same content as the 2001 version (now outdated),^39^ and only the wording was changed to simplify use and understanding.

## Conclusions

In this study, the number of GC-RCTs increased by year, but there was a significant burden of research waste. These findings suggest that there is room for improvement in research design, research implementation, results publication, and reuse of prospective data. This study may also provide evidence for points to be considered when developing future medical RCTs to improve experimental operations and reduce research waste.

## Notes

Supplement. **eTable 1.** Adjusted Logistic Regression Analysis of the Association of Key Study Characteristics With Publication Status **eTable 2.** Compliance With Items in Consolidated Standards of Reporting Trials (CONSORT) 2010 Checklist **eTable 3.** Characteristics of Randomized Clinical Trials by Reporting Adequacy **eTable 4.** Characteristics of Randomized Clinical Trials by Presence of Avoidable Design Flaws **eTable 5.** Characteristics of Randomized Clinical Trials by Presence of Guideline Citation **eTable 6.** Adjusted Logistic Regression Analysis of Association of Key Study Characteristics With Presence of Guideline Citation **eTable 7.** Characteristics of Randomized Clinical Trials by Reuse of Prospective Data **eTable 8.** Adjusted Logistic Regression Analysis of Association of Key Study Characteristics With Reuse of Prospective Data **eFigure 1.** Comparison of Randomized Clinical Trials Between Asian and Non-Asian Regions **eFigure 2.** Assessment of Risk of Bias

## References

[1] Fock KM. Review article: the epidemiology and prevention of gastric cancer. Aliment Pharmacol Ther. 2014;40(3):250-260. doi:10.1111/apt.12814 24912650

[2] Rawla P, Barsouk A. Epidemiology of gastric cancer: global trends, risk factors and prevention. Prz Gastroenterol. 2019;14(1):26-38. doi:10.5114/pg.2018.80001 30944675 PMC6444111

[3] Sakuramoto S, Sasako M, Yamaguchi T, ; ACTS-GC Group. Adjuvant chemotherapy for gastric cancer with S-1, an oral fluoropyrimidine. N Engl J Med. 2007;357(18):1810-1820. doi:10.1056/NEJMoa072252 17978289

[4] Cunningham D, Allum WH, Stenning SP, ; MAGIC Trial Participants. Perioperative chemotherapy versus surgery alone for resectable gastroesophageal cancer. N Engl J Med. 2006;355(1):11-20. doi:10.1056/NEJMoa055531 16822992

[5] Hu Y, Huang C, Sun Y, . Morbidity and mortality of laparoscopic versus open D2 distal gastrectomy for advanced gastric cancer: a randomized controlled trial. J Clin Oncol. 2016;34(12):1350-1357. doi:10.1200/JCO.2015.63.7215 26903580

[6] Yu J, Huang C, Sun Y, ; Chinese Laparoscopic Gastrointestinal Surgery Study (CLASS) Group. Effect of laparoscopic vs open distal gastrectomy on 3-year disease-free survival in patients with locally advanced gastric cancer: the CLASS-01 randomized clinical trial. JAMA. 2019;321(20):1983-1992. doi:10.1001/jama.2019.5359 31135850 PMC6547120

[7] Chapman SJ, Aldaffaa M, Downey CL, Jayne DG. Research waste in surgical randomized controlled trials. Br J Surg. 2019;106(11):1464-1471. doi:10.1002/bjs.11266 31393612

[8] Chandra R, Balachandar N, Wang S, Reznik S, Zeh H, Porembka M. The changing face of gastric cancer: epidemiologic trends and advances in novel therapies. Cancer Gene Ther. 2021;28(5):390-399. doi:10.1038/s41417-020-00234-z 33009508

[9] US National Library of Medicine. ClinicalTrials.gov. Accessed August 5, 2021. https://clinicaltrials.gov/

[10] Tse T, Fain KM, Zarin DA. How to avoid common problems when using ClinicalTrials.gov in research: 10 issues to consider. BMJ. 2018;361:k1452. doi:10.1136/bmj.k1452 29802130 PMC5968400

[11] Del Paggio JC, Berry JS, Hopman WM, . Evolution of the randomized clinical trial in the era of precision oncology. JAMA Oncol. 2021;7(5):728-734. doi:10.1001/jamaoncol.2021.0379 33764385 PMC7995135

[12] Shi C, Dumville JC, Cullum N, Rhodes S, McInnes E. Foam surfaces for preventing pressure ulcers. Cochrane Database Syst Rev. 2021;5:CD013621. doi:10.1002/14651858.CD013621.pub2 34097765 PMC8179968

[13] Janiaud P, Hemkens LG, Ioannidis JPA. Challenges and lessons learned from COVID-19 trials—should we be doing clinical trials differently? Can J Cardiol. 2021;S0828-282X(21)00285-3. doi:10.1016/j.cjca.2021.05.009 34077789 PMC8164884

[14] Gao J, Wei W, Wang G, Zhou H, Fu Y, Liu N. Circulating vitamin D concentration and risk of prostate cancer: a dose-response meta-analysis of prospective studies. Ther Clin Risk Manag. 2018;14:95-104. doi:10.2147/TCRM.S149325 29386901 PMC5767091

[15] Gautam S, Shrestha N, Mahato S, Nguyen TPA, Mishra SR, Berg-Beckhoff G. Diabetes among tuberculosis patients and its impact on tuberculosis treatment in South Asia: a systematic review and meta-analysis. Sci Rep. 2021;11(1):2113. doi:10.1038/s41598-021-81057-2 33483542 PMC7822911

[16] Fan Z, Li M, Chen X, . Prognostic value of cancer stem cell markers in head and neck squamous cell carcinoma: a meta-analysis. Sci Rep. 2017;7:43008. doi:10.1038/srep43008 28220856 PMC5318950

[17] Ioannidis JP. Effect of the statistical significance of results on the time to completion and publication of randomized efficacy trials. JAMA. 1998;279(4):281-286. doi:10.1001/jama.279.4.281 9450711

[18] Schulz KF, Altman DG, Moher D; CONSORT Group. CONSORT 2010 statement: updated guidelines for reporting parallel group randomised trials. BMJ. 2010;340:c332. doi:10.1136/bmj.c332 20332509 PMC2844940

[19] Boutron I, Moher D, Altman DG, Schulz KF, Ravaud P; CONSORT Group. Extending the CONSORT statement to randomized trials of nonpharmacologic treatment: explanation and elaboration. Ann Intern Med. 2008;148(4):295-309. doi:10.7326/0003-4819-148-4-200802190-00008 18283207

[20] Higgins JP, Altman DG, Gøtzsche PC, ; Cochrane Bias Methods Group; Cochrane Statistical Methods Group. The Cochrane Collaboration’s tool for assessing risk of bias in randomised trials. BMJ. 2011;343:d5928. doi:10.1136/bmj.d5928 22008217 PMC3196245

[21] Google. Google Scholar. Accessed May 5, 2021. https://scholar.google.com/

[22] Jari M, Shiari R, Salehpour O, Rahmani K. Epidemiological and advanced therapeutic approaches to treatment of uveitis in pediatric rheumatic diseases: a systematic review and meta-analysis. Orphanet J Rare Dis. 2020;15(1):41. doi:10.1186/s13023-020-1324-x 32019589 PMC7001204

[23] Xu BB, Lu J, Zheng ZF, . The predictive value of the preoperative C-reactive protein-albumin ratio for early recurrence and chemotherapy benefit in patients with gastric cancer after radical gastrectomy: using randomized phase III trial data. Gastric Cancer. 2019;22(5):1016-1028. doi:10.1007/s10120-019-00936-w 30739259

[24] Choi YY, Kim H, Shin SJ, . Microsatellite instability and programmed cell death-ligand 1 expression in stage II/III gastric cancer: post hoc analysis of the CLASSIC randomized controlled study. Ann Surg. 2019;270(2):309-316. doi:10.1097/SLA.0000000000002803 29727332

[25] Lu J, Tucciarone J, Padilla-Coreano N, He M, Gordon JA, Huang ZJ. Selective inhibitory control of pyramidal neuron ensembles and cortical subnetworks by chandelier cells. Nat Neurosci. 2017;20(10):1377-1383. doi:10.1038/nn.4624 28825718 PMC5614838

[26] Houghton C, Dowling M, Meskell P, . Factors that impact on recruitment to randomised trials in health care: a qualitative evidence synthesis. Cochrane Database Syst Rev. 2020;10:MR000045. doi:10.1002/14651858.MR000045.pub2 33026107 PMC8078544

[27] Liberati A. Need to realign patient-oriented and commercial and academic research. Lancet. 2011;378(9805):1777-1778. doi:10.1016/S0140-6736(11)61772-8 22098852

[28] Corner J, Wright D, Hopkinson J, Gunaratnam Y, McDonald JW, Foster C. The research priorities of patients attending UK cancer treatment centres: findings from a modified nominal group study. Br J Cancer. 2007;96(6):875-881. doi:10.1038/sj.bjc.6603662 17342090 PMC2360101

[29] Chalmers I, Bracken MB, Djulbegovic B, . How to increase value and reduce waste when research priorities are set. Lancet. 2014;383(9912):156-165. doi:10.1016/S0140-6736(13)62229-1 24411644

[30] Kim HH, Han SU, Kim MC, ; Korean Laparoendoscopic Gastrointestinal Surgery Study (KLASS) Group. Effect of laparoscopic distal gastrectomy vs open distal gastrectomy on long-term survival among patients with stage I gastric cancer: the KLASS-01 randomized clinical trial. JAMA Oncol. 2019;5(4):506-513. doi:10.1001/jamaoncol.2018.6727 30730546 PMC6459124

[31] Liu F, Huang C, Xu Z, ; Chinese Laparoscopic Gastrointestinal Surgery Study (CLASS) Group. Morbidity and mortality of laparoscopic vs open total gastrectomy for clinical stage I gastric cancer: the CLASS02 multicenter randomized clinical trial. JAMA Oncol. 2020;6(10):1590-1597. doi:10.1001/jamaoncol.2020.3152 32815991 PMC7441466

[32] Savović J, Jones HE, Altman DG, . Influence of reported study design characteristics on intervention effect estimates from randomized, controlled trials. Ann Intern Med. 2012;157(6):429-438. doi:10.7326/0003-4819-157-6-201209180-00537 22945832

[33] Giacalone NJ, Milani N, Rawal B, . Funding support and principal investigator leadership of oncology clinical trials using radiation therapy. Int J Radiat Oncol Biol Phys. 2018;102(1):34-43. doi:10.1016/j.ijrobp.2018.05.037 29970311

[34] Jairam V, Yu JB, Aneja S, Wilson LD, Lloyd S. Differences in funding sources of phase III oncology clinical trials by treatment modality and cancer type. Am J Clin Oncol. 2017;40(3):312-317. doi:10.1097/COC.0000000000000152 25374144

[35] Krzyzanowska MK, Pintilie M, Tannock IF. Factors associated with failure to publish large randomized trials presented at an oncology meeting. JAMA. 2003;290(4):495-501. doi:10.1001/jama.290.4.495 12876092

[36] Tian ZR, Yao M, Zhou LY, . Effect of docosahexaenoic acid on the recovery of motor function in rats with spinal cord injury: a meta-analysis. Neural Regen Res. 2020;15(3):537-547. doi:10.4103/1673-5374.266065 31571666 PMC6921345

[37] Correa AF, Jegede O, Haas NB, . Predicting renal cancer recurrence: defining limitations of existing prognostic models with prospective trial-based validation. J Clin Oncol. 2019;37(23):2062-2071. doi:10.1200/JCO.19.00107 31216227 PMC7085167

[38] Banno M, Tsujimoto Y, Kataoka Y. Studies registered in non-ClinicalTrials.gov accounted for an increasing proportion of protocol registrations in medical research. J Clin Epidemiol. 2019;116:106-113. doi:10.1016/j.jclinepi.2019.09.005 31521723

[39] Moher D, Schulz KF, Altman DG. The CONSORT statement: revised recommendations for improving the quality of reports of parallel-group randomised trials. Lancet. 2001;357(9263):1191-1194. doi:10.1016/S0140-6736(00)04337-3 11323066

