# Supplementary material for: Characteristics and Research Waste Among Randomized Clinical Trials in Gastric Cancer
Source: JAMA Netw Open. 2021 Sep 17;4(9):e2124760. doi: 10.1001/jamanetworkopen.2021.24760 (PMC8449283; doi:10.1001/jamanetworkopen.2021.24760)
Supplement: Supplement. — eTable 1. Adjusted Logistic Regression Analysis of the Association of Key Study Characteristics With Publication Status eTable 2. Compliance With Items in Consolidated Standards of Reporting Trials (CONSORT) 2010 Checklist eTable 3. Characteristics of Randomized Clinical Trials by Reporting Adequacy eTable 4. Characteristics of Randomized Clinical Trials by Presence of Avoidable Design Flaws eTable 5. Characteristics of Randomized Clinical Trials by Presence of Guideline Citation eTable 6. Adjusted Logistic Regression Analysis of Association of Key Study Characteristics With Presence of Guideline Citation eTable 7. Characteristics of Randomized Clinical Trials by Reuse of Prospective Data eTable 8. Adjusted Logistic Regression Analysis of Association of Key Study Characteristics With Reuse of Prospective Data eFigure 1. Comparison of Randomized Clinical Trials Between Asian and Non-Asian Regions eFigure 2. Assessment of Risk of Bias [file jamanetwopen-e2124760-s001.pdf]

Supplemental Online Content

Lu J, Xu B-b, Shen L-l, et al. Characteristics and research waste among randomized clinical trials in gastric cancer. *JAMA Netw Open*. 2021;4(9);e2124760.
doi:10.1001/jamanetworkopen.2021.24760

eTable 1. Adjusted Logistic Regression Analysis of the Association of Key Study Characteristics With Publication Status
eTable 2. Compliance With Items in Consolidated Standards of Reporting Trials (CONSORT) 2010 Checklist
eTable 3. Characteristics of Randomized Clinical Trials by Reporting Adequacy
eTable 4. Characteristics of Randomized Clinical Trials by Presence of Avoidable Design Flaws
eTable 5. Characteristics of Randomized Clinical Trials by Presence of Guideline Citation
eTable 6. Adjusted Logistic Regression Analysis of Association of Key Study Characteristics With Presence of Guideline Citation
eTable 7. Characteristics of Randomized Clinical Trials by Reuse of Prospective Data
eTable 8. Adjusted Logistic Regression Analysis of Association of Key Study Characteristics With Reuse of Prospective Data
eFigure 1. Comparison of Randomized Clinical Trials Between Asian and Non-Asian Regions
eFigure 2. Assessment of Risk of Bias

This supplemental material has been provided by the authors to give readers additional information about their work.

© 2021 Lu J et al. *JAMA Network Open.*

eTable 1. Adjusted Logistic Regression Analysis of the Association of Key Study Characteristics With Publication Status

	Univariate analysis			Multivariate analysis	
	OR (95%CI)	p value		OR (95%CI)	p value
Year of registration					
2000-2009	1.00				
After 2009	0.40 (0.20-0.81)	0.01		0.89 (0.35-2.27)	0.81
Intervention					
Pharmacological-related	1.00				
Non-pharmacological-related	0.56 (0.26-1.22)	0.14			
Region of PI					
Asian	1.00			1.00	
Non-Asian	2.76 (1.18-6.44)	0.02		2.76 (1.03-7.35)	0.04
Blinding					
None/open label	1.00				
Single	0.63 (0.20-2.04)	0.45			
Double or more	1.54 (0.69-3.46)	0.29			
Recruitment					
Monocentric	1.00			1.00	
Multicenter	6.89 (3.21-14.79)	<0.001		3.84 (1.58-9.38)	0.003
No. of participants					
< 200	1.00			1.00	
⩾200	3.90 (1.89-8.02)	<0.001		2.13 (0.90-5.05)	0.09
Funding					
None/departmental	1.00			1.00	
Industry/other external	5.06 (2.05-12.53)	<0.001		2.373 (1.05-5.38)	0.04

OR indicates odd ratio; CI, confidence interval; PI, principal investigator.

© 2021 Lu J et al. *JAMA Network Open.*

eTable 2. Compliance With Items in Consolidated Standards of Reporting Trials (CONSORT) 2010 Checklist			
**CONSORT Item**		**Pharmacological n=64**	**NPI n=17**
**1a**	Identification as a randomised trial in the title	49 (76.6%)	16 (94.1%)
**1b**	Structured summary of trial design, methods, results, and conclusions	62 (96.9%)	16 (94.1%)
**2a**	Scientific background and explanation of rationale	64 (100%)	17 (100%)
**2b**	Specific objectives or hypotheses	63 (98.4%)	17 (100%)
**3a**	Description of trial design (such as parallel, factorial) including allocation ratio	58 (90.6%)	11 (64.7%)
**3b***	Important changes to methods after trial commencement (such as eligibility criteria), with reasons	-	-
**4a**	Eligibility criteria for participants	63 (98.4%)	14 (82.4%)
**4b**	Settings and locations where the data were collected	45 (70.3%)	13 (76.5%)
**5**	The interventions for each group with sufficient details to allow replication	62 (96.9%)	15 (88.2%)
**5A****	Description of the components of the interventions and, if applicable, the procedure for individualizing treatment	N/A	14 (82.4%)
**5B****	Details of how the interventions were standardized	N/A	4 (23.5%)
**5C****	Details of how the adherence of care provers with the protocol was assessed or enhanced	N/A	1 (6%)
**6a**	Completely defined pre-specified primary and secondary outcome measures	60 (93.8%)	15 (88.2%)
**6b***	Any changes to trial outcomes after the trial commenced, with reasons	-	-
**7a**	How sample size was determined	58 (92.1%)	17 (100%)
**7b***	When applicable, explanation of any interim analyses and stopping guidelines	-	-
**8a**	Method used to generate the random allocation sequence	50 (78.1%)	11 (68.8%)

© 2021 Lu J et al. *JAMA Network Open.*

**8b**	Type of randomisation; details of any restriction (such as blocking and block size)	41 (64.1%)	10 (58.8%)
**9**	Mechanism used to implement the random allocation sequence	30 (46.9%)	6 (35.3%)
**10**	Who generated the random allocation sequence, enrolled participants, and assigned participants to interventions	29 (45.3%)	6 (35.3%)
**11a**	If done, who was blinded after assignment to interventions and how	55 (85.9%)	7 (41.2%)
**11b***	If relevant, description of the similarity of interventions	-	-
**12a**	Statistical methods used to compare groups for primary and secondary outcomes	56 (87.5%)	16 (94.1%)
**12b***	Methods for additional analyses, such as subgroup analyses and adjusted analyses	-	-
**13a**	The numbers of participants who were randomised, received treatment, and analysed for the primary outcome	62 (96.9%)	16 (94.1%)
**13b**	For each group, losses and exclusions after randomisation, together with reasons	63 (98.4%)	16 (94.1%)
**14a**	Dates defining the periods of recruitment and follow-up	61 (95.3%)	16 (94.1%)
**14b***	Why the trial ended or was stopped	-	-
**15**	A table showing baseline demographic and clinical characteristics for each group	64 (100%)	17 (100%)
**16**	For each group, number of participants analysed and whether the analysis was by original assigned groups	63 (98.4%)	17 (100%)
**17a**	For each primary and secondary outcome, results for each group, and the estimated effect size and its precision	53 (82.8%)	6 (35.3%)
**17b**	For binary outcomes, presentation of both absolute and relative effect sizes is recommended	32 (50.0%)	4 (23.5%)
**18***	Results of any other analyses performed, including subgroup analyses and adjusted analyses	-	-
**19**	All important harms or unintended effects in each group	62 (96.9%)	12 (70.6%)

© 2021 Lu J et al. *JAMA Network Open.*

**20**	Trial limitations, addressing sources of potential bias, imprecision, and, if relevant, multiplicity of analyses	32 (50.0%)	10 (58.8%)
**21**	Generalisability (external validity, applicability) of the trial findings	52 (81.3%)	7 (41.2%)
**22**	Interpretation consistent with results, balancing benefits and harms, and considering other relevant evidence	61 (95.3%)	16 (94.1%)
**23**	Registration number and name of trial registry	63 (98.4%)	14 (82.4%)
**24**	Where the full trial protocol can be accessed, if available	20 (31.3%)	5 (29.4%)
**25**	Sources of funding and other support (such as supply of drugs), role of funders	58 (90.6%)	10 (58.8%)
*A conditional item for which not all manuscripts were scored			
** items relate to non-pharmacological (NPI) RCTs only			

© 2021 Lu J et al. *JAMA Network Open.*

**eTable 3.** Characteristics of Randomized Clinical Trials by Reporting Adequacy			
	Adequate reporting, No. (%)	Inadequate reporting, No. (%)	p-value
Year of publication			0.214
2000-2009	7 (10.8)	4 (25.0)	
After 2009	58 (89.2)	12 (75.0)	
Phase			0.696
3	56 (86.2)	13 (81.3)	
4	9 (13.8)	3 (18.8)	
Intervention			0.004
Pharmacological-related	56 (86.2)	8 (50.0)	
Non-pharmacological-related	9 (13.8)	8 (50.0)	
Region of PI			0.755
Asian	42 (64.6)	11 (68.8)	
Non-Asian	23 (35.4)	5 (31.3)	
No. of arms			0.610
Two arms	62 (95.4)	16 (100.0)	
Multiarm	3 (4.6)	0 (0.0)	
Blinding			0.679
None/open label	40 (61.5)	10 (62.5)	
Single	4 (6.2)	2 (12.5)	
Double or more	21 (32.3)	4 (25.0)	
Recruitment			0.032
Monocentric	15 (23.1)	8 (50.0)	
Multicenter	50 (76.9)	8 (50.0)	
No. of participants			0.011
<200	14 (21.5)	9 (56.3)	
≥200	51 (78.5)	7 (43.8)	
Funding			0.125
None/departmental	35 (53.8)	12 (75.0)	
Industry/other external	30 (46.2)	4 (25.0)	
PI: principal investigator.			

© 2021 Lu J et al. *JAMA Network Open.*

**eTable 4.** Characteristics of Randomized Clinical Trials by Presence of Avoidable Design Flaws			
	Absence of design flaw, No. (%)	Presence of design flaw, No. (%)	p-value
Year of registration			0.016
2000-2009	6 (33.3)	41 (65.1)	
After 2009	12 (66.7)	22 (34.9)	
Phase			0.283
3	17 (94.4)	52 (82.5)	
4	1 (5.6)	11 (17.5)	
Intervention			0.101
Pharmacological-related	17 (94.4)	47 (74.6)	
Non-pharmacological-related	1 (5.6)	16 (25.4)	
Region of PI			0.662
Asian	11 (61.1)	42 (66.7)	
Non-Asian	7 (38.9)	21 (33.3)	
No. of arms			0.588
Two arms	18 (100.0)	60 (95.2)	
Multiarm	0 (0.0)	3 (4.8)	
Blinding			0.059
None/open label	7 (38.9)	43 (68.3)	
Single	2 (11.1)	4 (6.3)	
Double or more	9 (50.0)	16 (25.4)	
Recruitment			0.211
Monocentric	3 (16.7)	20 (31.7)	
Multicenter	15 (83.3)	43 (48.3)	
No. of participants			0.015
<200	1 (5.6)	11 (17.5)	
≥200	17 (94.4)	52 (82.5)	
Funding			0.062
None/departmental	7 (38.9)	40 (63.5)	
Industry/other external	11 (61.1)	23 (36.5)	
PI: principal investigator.			

© 2021 Lu J et al. *JAMA Network Open.*

**eTable 5.** Characteristics of Randomized Clinical Trials by Presence of Guideline Citation			
	Presence of citing by guidelines, No. (%)	Absence of citing by guidelines, No. (%)	p-value
Region of PI			0.677
Asian	24 (68.6)	23 (63.9)	
Non-Asian	11 (31.4)	13 (36.1)	
Intervention			0.101
Pharmacological-related	30 (85.7)	25 (69.4)	
Non-pharmacological-related	5 (14.3)	11 (30.6)	
No. of arms			1.000
Two arms	35 (100.0)	35 (97.2)	
Multiarm	0 (0.0)	1 (2.8)	
Blinding			0.869
None/open label	23 (65.7)	21 (58.3)	
Single	3 (8.6)	3 (8.3)	
Double or more	9 (25.7)	12 (33.3)	
Recruitment			0.048
Monocentric	7 (20.0)	15 (41.7)	
Multicenter	28 (80.0)	21 (58.3)	
No. of participants			<0.001
< 200	4 (11.4)	19 (52.8)	
⩾200	31 (88.6)	17 (47.2)	
Positive results			0.001
Absence	8 (22.9)	22 (61.1)	
Presence	27 (77.1)	14 (38.9)	
Funding			0.288
None/departmental	17 (48.6)	22 (61.1)	
Industry/other external	18 (51.4)	14 (38.9)	
PI indicates principal investigator.			

© 2021 Lu J et al. *JAMA Network Open.*

**eTable 6.** Adjusted Logistic Regression Analysis of Association of Key Study Characteristics With Presence of Guideline Citation							
	Univariate analysis				Multivariate analysis		
	OR	95%CI	p-value		OR	95%CI	p-value
Intervention							
Pharmacological-related	1.000						
Non-pharmacological-related	0.379	0.116-1.236	0.108				
Region of PI							
Asian	1.000						
Non-Asian	0.811	0.303-2.173	0.677				
Blinding							
None/open label	1.000						
Single	0.913	0.166-5.029	0.917				
Double or more	0.685	0.240-1.952	0.479				
Recruitment							
Monocentric	1.000						
Multicenter	2.857	0.989-8.252	0.052				
No. of participants							
<200	1.000				1.000		
≥200	8.662	2.532-29.628	0.001		13.545	3.295-55.675	<0.001
Positive results							
Absence	1.000				1.000		
Presence	5.304	1.884-14.932	0.002		8.419	2.416-29.341	0.001
Funding							
None/departmental	1.000						
Industry/other external	1.664	0.648-4.270	0.290				
OR indicates odd ratio; PI, principal investigator.							

© 2021 Lu J et al. *JAMA Network Open.*

**eTable 7.** Characteristics of Randomized Clinical Trials by Reuse of Prospective Data			
	Presence of reuse of prospective data, No. (%)	Absence of reuse of prospective data, No. (%)	p-value
Region of PI			0.034
Asian	8 (44.4)	45 (71.4)	
Non-Asian	10 (55.6)	18 (28.6)	
Intervention			0.101
Pharmacological-related	17 (94.4)	47 (74.6)	
Non-pharmacological-related	1 (5.6)	16 (25.4)	
No. of arms			0.586
Two arms	18 (100.0)	60 (95.2)	
Multiarm	0 (0.0)	3 (4.8)	
Blinding			0.703
None/open label	10 (55.6)	40 (63.5)	
Single	1 (5.6)	5 (7.9)	
Double or more	7 (38.9)	18 (28.6)	
Recruitment			0.015
Monocentric	1 (5.6)	22 (34.9)	
Multicenter	17 (94.4)	41 (65.1)	
No. of participants			0.065
<200	2 (11.1)	21 (33.3)	
≥200	16 (88.9)	42 (66.7)	
Positive results			0.032
Absence	3 (16.7)	28 (44.4)	
Presence	15 (83.3)	35 (55.6)	
Funding			0.062
None/departmental	7 (38.9)	40 (63.5)	
Industry/other external	11 (61.1)	23 (36.5)	
PI: principal investigator.			

© 2021 Lu J et al. *JAMA Network Open.*

eTable 8. Adjusted Logistic Regression Analysis of Association of Key Study Characteristics With Reuse of Prospective Data

	Univariate analysis				Multivariate analysis		
	OR	95%CI	p-value		OR	95%CI	p-value
Intervention							
Pharmacological-related	1.000						
Non-pharmacological-related	0.169	0.021–1.375	0.096				
Region of PI							
Asian	1.000				1.000		
Non-Asian	3.309	1.119–9.785	0.031		3.447	1.103–10.773	0.033
Blinding							
None/open label	1.000						
Single	0.780	0.082–7.450	0.829				
Double or more	1.517	0.497–4.629	0.464				
Recruitment							
Monocentric	1.000				1.000		
Multicenter	9.350	1.165–75.052	0.035		9.723	1.179–80.218	0.035
Positive results							
Absence	1.000				1.000		
Presence	4.000	1.052-15.207	0.042		8.513	1.786-40.578	0.007
No. of participants							
<200	1.000						
≥200	3.810	0.798–18.191	0.094				
Funding							
None/departmental	1.000						
Industry/other external	2.857	0.969–8.422	0.057				
*The primary endpoints reported were as assumed.							
OR indicates odd ratio; PI, principal investigator.							

© 2021 Lu J et al. *JAMA Network Open.*

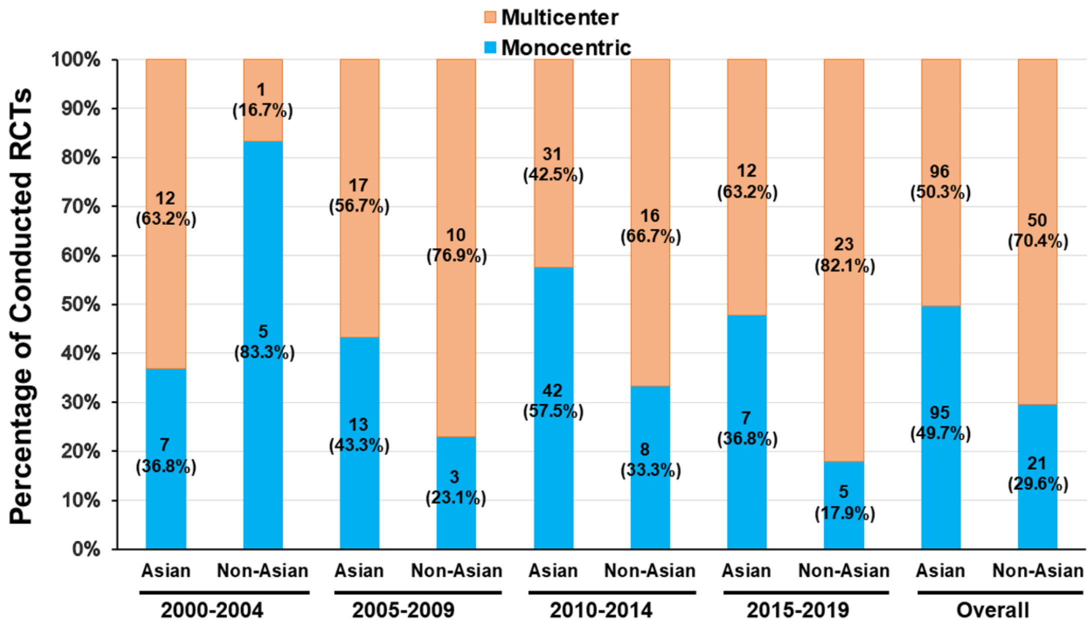

eFigure 1. Comparison of Randomized Clinical Trials Between Asian and Non-Asian Regions

© 2021 Lu J et al. *JAMA Network Open.*

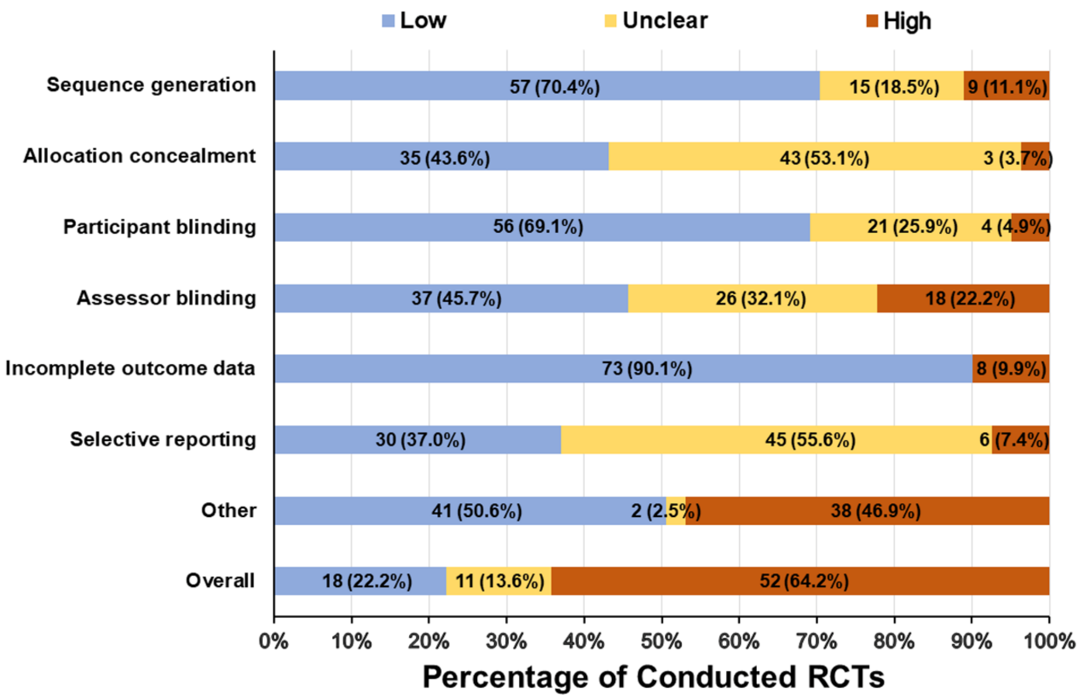

eFigure 2. Assessment of Risk of Bias

© 2021 Lu J et al. *JAMA Network Open.*
